# Effect of Short-Term Supplementation with Ready-to-Use Therapeutic Food or Micronutrients for Children after Illness for Prevention of Malnutrition: A Randomised Controlled Trial in Uganda

**DOI:** 10.1371/journal.pmed.1001951

**Published:** 2016-02-09

**Authors:** Saskia van der Kam, Stephanie Roll, Todd Swarthout, Grace Edyegu-Otelu, Akiko Matsumoto, Francis Xavier Kasujja, Cristian Casademont, Leslie Shanks, Nuria Salse-Ubach

**Affiliations:** 1 Médecins Sans Frontières, Amsterdam, Netherlands; 2 Ecole de Santé Publique, Centre de Recherche en Politiques et Systèmes de Santé-Santé Internationale, Université Libre de Bruxelles, Brussels, Belgium; 3 Institute for Social Medicine, Epidemiology and Health Economics, Charité-Universitätsmedizin, Berlin, Germany; 4 Ministry of Health of Uganda, Kampala, Uganda; 5 Médecins Sans Frontières, Barcelona, Spain; Makerere University Medical School, UGANDA

## Abstract

**Background:**

Globally, Médecins Sans Frontières (MSF) treats more than 300,000 severely malnourished children annually. Malnutrition is not only caused by lack of food but also by illnesses and by poor infant and child feeding practices. Breaking the vicious cycle of illness and malnutrition by providing ill children with nutritional supplementation is a potentially powerful strategy for preventing malnutrition that has not been adequately investigated. Therefore, MSF investigated whether incidence of malnutrition among ill children <5 y old could be reduced by providing a fortified food product or micronutrients during their 2-wk convalescence period. Two trials, one in Nigeria and one in Uganda, were conducted; here, we report on the trial that took place in Kaabong, a poor agropastoral region of Karamoja, in east Uganda. While the region of Karamoja shows an acute malnutrition rate between 8.4% and 11.5% of which 2% to 3% severe malnutrition, more than half (58%) of the population in the district of Kaabong is considered food insecure.

**Methods and Findings:**

We investigated the effect of two types of nutritional supplementation on the incidence of malnutrition in ill children presenting at outpatient clinics during March 2011 to April 2012 in Kaabong, Karamoja region, Uganda, a resource-poor region where malnutrition is a chronic problem for its seminomadic population. A three-armed, partially-blinded, randomised controlled trial was conducted in children diagnosed with malaria, diarrhoea, or lower respiratory tract infection. Non-malnourished children aged 6 to 59 mo were randomised to one of three arms: one sachet/d of ready-to-use therapeutic food (RUTF), two sachets/d of micronutrient powder (MNP), or no supplement (control) for 14 d for each illness over 6 mo. The primary outcome was the incidence of first negative nutritional outcome (NNO) during the 6 mo follow-up. NNO was a study-specific measure used to indicate progression to moderate or severe acute malnutrition; it was defined as weight-for-height z-score <−2, mid-upper arm circumference (MUAC) <115 mm, or oedema, whichever came first.

Of the 2,202 randomised participants, 51.2% were girls, and the mean age was 25.2 (±13.8) mo; 148 (6.7%) participants were lost to follow-up, 9 (0.4%) died, and 14 (0.6%) were admitted to hospital. The incidence rates of NNO (first event/year) for the RUTF, MNP, and control groups were 0.143 (95% confidence interval [CI], 0.107–0.191), 0.185 (0.141–0.239), and 0.213 (0.167–0.272), respectively. The incidence rate ratio was 0.67 (95% CI, 0.46–0.98; *p* = 0.037) for RUTF versus control; a reduction of 33.3%. The incidence rate ratio was 0.86 (0.61–1.23; *p* = 0.413) for MNP versus control and 0.77 for RUTF versus MNP (95% CI 0.52–1.15; *p* = 0.200). The average numbers of study illnesses for the RUTF, MNP, and control groups were 2.3 (95% CI, 2.2–2.4), 2.1 (2.0–2.3), and 2.3 (2.2–2.5). The proportions of children who died in the RUTF, MNP, and control groups were 0%, 0.8%, and 0.4%.

The findings apply to ill but not malnourished children and cannot be generalised to a general population including children who are not necessarily ill or who are already malnourished.

**Conclusions:**

A 2-wk nutrition supplementation programme with RUTF as part of routine primary medical care to non-malnourished children with malaria, LRTI, or diarrhoea proved effective in preventing malnutrition in eastern Uganda. The low incidence of malnutrition in this population may warrant a more targeted intervention to improve cost effectiveness.

**Trial Registration:**

clinicaltrials.gov NCT01497236

## Abstract 2

A clinical trial set in Uganda shows that short-term supplementation with ready-to-use food in children following a bout of acute illness can prevent malnutrition. This short term measure has longer term effects in reducing morbidity in a vulnerable population.

## Editors' Summary

**Background:**

Globally, malnutrition—poor nutrition—is thought to contribute to nearly half of all child deaths. Malnutrition can be chronic or acute. Chronic (long-term) malnutrition causes stunting. A child who is stunted has a low height for his or her age when compared to WHO Child Growth Standards, which chart the growth of a reference population. By contrast, acute malnutrition causes wasting. A wasted child has a low weight for his or her height. Malnutrition can be caused by not having enough to eat, by not eating enough of the right foods, or being unable to use the food that one does eat. In many tropical countries, recurrent infections are also an important cause of malnutrition among children. Diarrhea, lower respiratory tract infections, and malaria all have a negative effect on the growth of children. Importantly, inadequate nutrition limits recovery from infection, thereby setting up a vicious cycle of illness and malnutrition.

**Why Was This Study Done?:**

It might be possible to reduce the global burden of malnutrition among children by breaking this vicious cycle. One way to do this might be to provide ill children with a nutritional supplement such as RUTF or a MNP. RUTF—a nutrient supplement based on peanut butter mixed with dried skim milk, vitamins, and minerals—is a paste that can be eaten directly. Micronutrients are vitamins and minerals that everyone needs in small quantities for good health; MNP is added to porridge or other meals. In this randomized controlled trial undertaken by MSF, a not-for-profit organization that delivers emergency medical aid worldwide, the researchers investigate whether short-term provision of RUTF or MNP prevents the development of malnutrition among ill children under 5 y old living in Kaabong, a poor agropastoral region in eastern Uganda, where about 10% of children are acutely malnourished.

**What Did the Researchers Do and Find?:**

The researchers randomly assigned 2,202 non-malnourished children who visited outpatient clinics in Kaabong with malaria, diarrhea or lower respiratory tract infection to one of three trial arms. Children assigned to the two intervention arms were given RUTF or MNP by their caregivers for 14 d following each illness over a 6-mo period. Children assigned to the control arm received no supplement. The primary outcome of the trial was the incidence of the first NNO—a weight-for-height z-score below −2 (a score that compares a child’s weight-for-height with that of a reference population; a z-score of −2 or less indicates acute malnutrition), a MUAC of less than 115 mm, or nutritional edema (swelling caused by malnutrition)—during follow-up (the incidence of a condition is the proportion of a population affected by that condition during a specified time period). The incidence rates of NNO were 0.143, 0.185, and 0.213 first events/year observation in the RUTF, MNP, and control groups, respectively. Notably, the IRR of NNO for RUTF versus control was 0.67, a significant reduction in the incidence of malnutrition in the RUTF group of 33% compared with the control group (a significant reduction is unlikely to have occurred by chance). By contrast, supplementation with NMP did not significantly reduce the incidence of malnutrition.

**What Do These Findings Mean?:**

These findings show that, among non-malnourished children with malaria, lower respiratory tract infection, or diarrhea living in Kaabong, Uganda, provision of an RUTF-based nutritional supplement for 14 d following an illness as part of routine primary medical care prevented malnutrition. Because this trial only enrolled children who were non-malnourished, these findings cannot be generalized to all ill children with an infectious illness in Kaabong or similar settings—many ill children presenting at outpatient clinics are acutely malnourished. Interestingly, a companion trial undertaken by MSF in Goronyo, Nigeria found no reduction in the incidence of malnutrition among non-malnourished and moderately acutely malnourished children following short-term supplementation with either RUTF or MNP. The researchers suggest that the different results in the two trials may reflect the higher incidence of malnutrition and illness in Goronyo compared to Kaabong. Indeed, given the low incidence of malnutrition in Kaabong, the researchers suggest that a more targeted intervention such as only providing RUTF to ill children younger than 3 y old might be more cost-effective than providing nutritional supplementation to all ill children in Kaabong and similar settings.

**Additional Information:**

This list of resources contains links that can be accessed when viewing the PDF on a device or via the online version of the article at http://dx.doi.org/10.1371/journal.pmed.1001951.

- A *PLOS Medicine* Research Article by van der Kam et al. describing the companion trial investigating the effect of short-term food supplementation for children in Nigeria after illness on the incidence of malnutrition is available
- More information about this trial is available
- The MSF website contains information about malnutrition around the world; "Starved for Attention" is an international multimedia campaign launched in 2010 by MSF and the VII Photo agency to rewrite the story of childhood malnutrition
- The not-for-profit organization UNICEF, which protects the rights of children and young people around the world, provides detailed information on nutrition among children and statistics on malnutrition among children; a short 2013 article describes UNICEF efforts to reduce malnutrition in Uganda
- The WHO Child Growth Standards are available (in several languages)
- The World Food Programme is the world’s largest humanitarian agency fighting hunger worldwide; its website provides information about hunger and malnutrition in Uganda
- The Emergency Nutrition Network (ENN) is an interactive website for knowledge sharing and peer support to strengthen the evidence and know-how for effective nutrition interventions in countries prone to crisis and high levels of malnutrition
- The International Lipid-based Nutrient Supplements (iLiNS) project aims to help prevent malnutrition by developing Lipid-based Nutrient Supplements and test their efficiency and by collecting and sharing publications on LNS.

## Introduction

Treatment of malnutrition is an important part of the medical programmes of Médecins Sans Frontières (MSF). In 2011, the year the research presented here was conducted, MSF treated 348,017 severely malnourished children and 59,984 moderately malnourished children worldwide. It is imperative that MSF explores appropriate ways to prevent malnutrition, of which an effective strategy might be providing a nutritional supplementation to ill children.

The cause of malnutrition in most tropical countries is multifactorial, involving not only an inadequate diet but also recurrent infections [1,2]. When inadequate nutrition limits recovery, the risk of a permanently lowered nutritional status is increased [3], as is the risk of mortality due to illness [4–6]. There is extensively documented evidence of growth faltering related to diarrhoea [7–9]. Furthermore, malaria episodes have been associated with malnutrition, and malnutrition weakens the immune system and is thus associated with a more severe outcome of a malaria infection [10–13]. Lower respiratory tract infections (LRTIs) have also shown a negative effect on growth [2,8].

Acute weight loss during episodes of acute infection can be mitigated by good nutrition. Studies of supplementation for patients with diarrhoea [14,15] have shown that nutritional supplementation immediately results in lower weight loss. Supplementation for patients with respiratory tract infection and malaria was also studied, and these studies concluded that dietary supplementation would increase weight gain during convalescence [16,17].

A research in the Democratic Republic of Congo showed that children with malaria gained weight faster during the period of supplementation when given 14 d of ready-to-use therapeutic food (RUTF), though this difference was not seen at the 4 wk follow-up [17].

Most studies that show a positive effect of nutritional supplementation in convalescence used a fortified food such as enriched porridges or lipid-based fortified foods such as RUTF [14,17]. Micronutrients also reduce the convalescence time [18–23], although some studies did not find an effect of multi-micronutrients on growth during convalescence of ill children [24,25]. As multi-micronutrients are cheaper than a porridge or a fortified food, it is necessary to investigate whether fortified food or micronutrients alone could promote an improved nutritional status during convalescence.

The World Health Organization (WHO) recommends that caretakers give ill children additional nutritious meal daily during recovery up to 2 wk after the onset of illness [26–28]. In resource-poor settings, the typical context of MSF’s programmes, this strategy is likely to be ineffective, as caregivers often lack healthy ingredients and other resources to implement this recommendation. A more effective strategy to reduce disease-related malnutrition in resource-poor areas may be to provide an ill child with a nutritional supplement at the point of care, the outpatient health clinic. Therefore, MSF investigated whether a supplementation of either a lipid-based fortified food or micronutrients for 2 wk after an illness is effective in reducing the incidence of malnutrition over a period of 6 mo. Two trials, one in Nigeria and one in Uganda, were conducted; here, we report on the trial that took place in Uganda, while the trial in Nigeria is reported elsewhere [29].

## Methods

### Approval

The trial was registered at clinicaltrials.gov number NCT01497236. The full protocol and the statistical analysis plan (SAP) can be accessed in the supporting information files (S1 Text and S2 Text). Ethical approval was obtained from the Uganda National Council of Science and Technology (UNCST) on 20th December 2010 and an extension for the study granted on 11th November 2011. The MSF Ethical Review Board granted approval for the study on 12th December, 2010.

### Setting

Kaabong is a remote and scarcely populated district in the Karamoja region of Uganda. Socioeconomic infrastructure, health services, and facilities—such as schools, health centres, and potable water—are weak, and the region has faced long periods of conflict and insecurity. This makes Karamoja one of the least developed parts of the country.

Karamoja is semiarid and has only one rainy season from early April to May. The agropastoral population of Kaabong grow sorghum, maize, millet, groundnuts, sunflower, cowpeas, and beans. They keep goats and sheep—better-off groups tend to own cattle. Nearly half the households depend on borrowing and exchange and food assistance [30,31]. Food security is fragile as a result of poverty and cycles of drought and floods. In 2009, 58.4% of the Kaabong population was considered to be food insecure [31]. The food insecurity was exacerbated by ongoing conflict that ended during the implementation of the study in 2011. During the study period, overall household food security showed improvement in the entire region.

As a result, during the study period, the prevalence of malnutrition was generally not high. The nutrition surveillance system gave a prevalence of acute malnutrition of 8.5% in May 2011, 8.4% in December 2011, and 11.5% in May 2012; of this, 2% to 3% was severe acute malnutrition [32].

In an analysis of 2014, the UN World Food Program concluded that the poor nutritional status among children in Karamoja was the result of a combination of high incidence of fever, malaria, and diarrhoea, poor sanitation facilities, lack of vitamin A supplementation, and a poor dietary diversity, rather than a lack of food per se [33].

The involvement of MSF in Kaabong started in 2007 with support to mother and child care, including a feeding programme to respond to a nutritional crisis caused by a severe drought. Because of major improvements in the health situation, MSF withdrew its support to the health activities completely in December 2012, and only this study was continued until completion.

During the study, many households (37.3%) received income through food aid cash for work programmes. A targeted food distribution programme to extremely vulnerable households became operational in March 2012. In addition, a supplementary feeding programme for treatment of moderately malnourished children (supported by World Vision International) and a therapeutic feeding program for treatment of severely malnourished children (supported by Action Contre le Faim) were present.

### Study Objectives and Endpoints

The aim of the three-armed, partially-blinded, randomised controlled trial was to investigate the effectiveness of 14 d nutritional supplementation with RUTF or a multi-micronutrient powder (MNP) given to non-malnourished children aged 6–59 mo and diagnosed with and treated for malaria, diarrhoea, and/or LRTIs in reducing the incidence of acute malnutrition during a follow-up time of 6 months. Given the complexity of malnutrition in the trial’s study population, a study-specific primary endpoint event was compiled. This is known as a negative nutritional outcome (NNO) and defined as weight-for-height z-score <−2, mid-upper arm circumference (MUAC) <115 mm, or nutritional oedema, whichever came first. Secondary outcomes included changes in anthropometric indicators, morbidity, and mortality.

### Study Population and Randomisation

Non-malnourished children (weight-for-height z-score >−2, MUAC >125 mm, and absence of nutritional oedema) aged 6–59 mo diagnosed at the regular outpatient clinic with one or more of the three study diseases (malaria, diarrhoea, LRTI) were included in the study when living within approximately 60 min walking distance from the clinic and intending to stay in the area during the 6-mo follow-up.

Children who met the criteria for severe acute malnutrition (defined as weight-for-height z-score <−3, MUAC <115 mm, or nutritional oedema) or moderate acute malnutrition (defined as weight-for-height z-score between −3 and −2, or MUAC 115–125 mm), were exclusively breastfed, had a severe disease (for example, severe malaria, severe pneumonia, or severe anaemia), needed admission to hospital, had a sibling enrolled in the study, or were offspring of staff of the study were excluded.

Children were randomised in a 1:1:1 ratio to one of three intervention groups. During the 6-mo follow-up, children in the RUTF and MNP groups received nutritional supplements for 14 d whenever they were diagnosed with at least one of the three study diseases, with a maximum of 14 d of supplementation in any 28-d period. The MNP (MixMe, DSM Ltd, Switzerland) contained only micronutrients according the UN formulation [34]. Children in the MNP group received two doses per day, to ensure a micronutrient composition comparable to one sachet of RUTF (Plumpynut, Nutriset, France) (Table 1). The caretakers were instructed to mix MNP in the meal (for example, porridge) just before consumption. All groups (including the control group) received health education, including the message that following an illness, a child should eat one extra, healthy meal per day for 2 wk.

**Table 1:** Nutritional supplements’ composition per serving.

Nutrients	1 sachet RUTF^1^	2 sachets MNP^2^
Energy (kcal)	500	
Protein (g; % total energy)	11.6; 10%	
Lipid (g; % total energy)	29.5; 56%	
Calcium (mg)	276	
Phosphorus (mg)	276	
Potassium (mg)	1,022	
Magnesium (mg)	84.6	
Zinc (mg)	12.9	8.2
Copper (mg)	1.6	1.1
Iron (mg)	10.6	20.0
Iodine (μg)	96	180
Selenium (μg)	27.6	34.0
Sodium (μg)	<267	
Vitamin A (μg)	840	800
Vitamin D (μg)	15	10
Vitamin E (μg)	18.4	10.0
Vitamin C (mg)	49	60
Thiamine (mg)	0.55	1.0
Riboflavin (mg)	1.66	1.0
Niacin (mg)	4.88	12.0
Pyridoxine (mg)	0.55	1.0
Cobalamin (μg)	1.7	1.8
Folic Acid (μg)	193	300
Vitamin K (μg)	19.3	
Biotin (μg)	60	
Pantothenic Acid (mg)	2.85	

MNP: micronutrient powder; RUTF: ready-to-use therapeutic food.

^1^Sachet contains 92 g.

^2^2 sachets contain 2 g.

The sample size was based on the assumption of a baseline/control group incidence rate of first NNO event of 0.20 within 6 mo. A 30% reduction, considered a clinically and operationally relevant improvement, would result in a targeted incidence rate of 0.14 events in 6 mo in each of the two treatment groups. Using a Poisson regression model, 80% power at a 0.05 significance level, and an assumed drop-out rate of approximately 10%, a sample size of 734 children was needed in each group (2,202 in total for all three groups).

Simple randomisation was based on a computer-generated randomisation list made by an expert independent of the study. Participants entering the study received a study number, and only the staff members physically giving the supplement were able to connect the study number to the treatment group after all other procedures, including collection of baseline data, were finalised. All other study staff (including clinicians diagnosing illnesses and deciding on giving an allocation) were blinded to the allocation treatment throughout the study. The statistician was unblinded after the completion of the analysis.

### Procedures

Patients were screened in the regular outpatient clinic on eligibility, and potential participants were referred to the adjacent study clinic for further inclusion procedures. Activities at inclusion included provision of information and obtaining written consent of caretakers; in case the caretaker was illiterate, the consent form was to be read out loud, and consent was given by thumbprint in the presence of a witness. The enrolled participants were followed after the first 14 d and then monthly during 6 mo. If a child was ill, they were invited to come whenever they felt that was needed. At each visit, the health and nutritional status of the participant was assessed.

Anthropometric measurements—weight, height, oedema, and MUAC—were taken at every visit to the study clinic. Two types of electronic weighing scales were used to measure weight: SECA model 354, with a precision of 10–20 g, and SECA 869, a mother/baby scale with a precision of 100 g. Length or height (change of measuring position at 85 cm of height) was measured using a precision height board (infant–child–adult measuring board ICAM, aluminium, precision 1 mm, Promes). MUAC was measured using a standard MUAC tape (MSF, precision 2 mm).

Malaria was diagnosed by conducting a malaria rapid diagnostic test (RDT; SD-bioline Combo Standard Diagnostic, Korea). All new participants were tested on enrollment; during the study, participants would have a malaria test on indication (fever or history of fever).

Diarrhoea was defined as three or more loose stools (bloody or nonbloody) per 24 h and was diagnosed by mothers’ report. LRTI was diagnosed by the following algorithm: children with cough or having difficulty breathing, an increased respiratory rate (>50 breaths/min for children aged 6–11 mo old and >40 breaths/minute for children aged 12–59 mo), and absence of chest in-drawing were diagnosed as nonsevere LRTI; chest in-drawing was considered a sign of severe LRTI, needing referral to the hospital.

All children received standard care and treatment according to current national medical protocols. Uncomplicated malaria was treated for 3 d with a six-dose regimen of Artemether–Lumefantrine (Coartem). Acute watery diarrhoea was treated using oral rehydration therapy with low osmolarity oral rehydration salts and zinc according to WHO guidelines, regardless of the supplement received. LRTI was treated with amoxicillin 75 mg/kg/d for 5–7 d. The first treatment was given under medical supervision, and the use of and need for the drug were explained to the mother. At every visit, health education was provided, including prevention and treatment of malaria and diarrhoea and the advice to give an ill child an extra nutritious meal for 14 d. Participants presenting with other nonsevere diseases were treated in the study clinic according to the national protocol.

Home visitors supported the study by reminding the caretakers to come to the appointments, urging absentees to return, and reporting on deaths that occurred at home. Any death was immediately reported and reviewed by a national doctor, principal investigator, epidemiologist, and medical director of MSF.

Compliance was measured by questionnaires, asking how many times and for how many days the participant consumed the supplement. In addition, the caretaker was asked to return full or empty sachets of each distribution. In March, June, and September 2012, three focus group discussions were held with caretakers who had completed the trial to discuss the supplements and medication, compliance, and any barriers regarding use of the supplements.

### Data Analysis

The primary outcome (rates of first NNO) among the treatment groups was analysed by a Poisson regression model including the three intervention groups with contrasts for each two-group comparison. As there were three interventional groups to be compared, a hierarchical test procedure was used to account for multiplicity. If a significant result (significance level 0.05 two-sided) was observed in the RUTF group compared with control, MNP was then compared with control; if this was also statistically significant at 0.05 two-sided, RUTF was subsequently compared with MNP in a noninferiority approach with a noninferiority margin of 2% (that is, allowing a slightly worse result in the MNP compared with the RUTF group, statistically significant at 0.025 one-sided). If no significant result was observed in the first or second step, all other comparisons were considered exploratory.

The incidence rate of malnutrition (NNO) is expressed in the first event per 365 observation d (events/y) and is extrapolated from the study period of 168 d (24 wk).

A priori defined subgroups were analysed by including interaction terms in the main model. Secondary outcomes were analysed using logistic regression or analysis of covariance, with treatment group and baseline values as covariates.

Any participant attending at least the first and the last visit was counted as having completed the study. A participant developing acute malnutrition (weight-for-height z-score <−2, MUAC <115, or nutritional oedema) was categorised as an NNO and referred to a feeding programme. Participants who were developing a NNO, seriously ill participants, patients needing admission to hospital, and participants with measles were withdrawn and their data up to the moment of withdrawal was used.

When a serious protocol violation occurred or the participant was erroneously included, the participant was withdrawn and the data was not used. The primary outcomes were also analysed for a per protocol population that excluded participants if they had abandoned the study, had been admitted to hospital, had a low compliance, or had measles.

## Results

### Participant Flow

**Fig 1:**
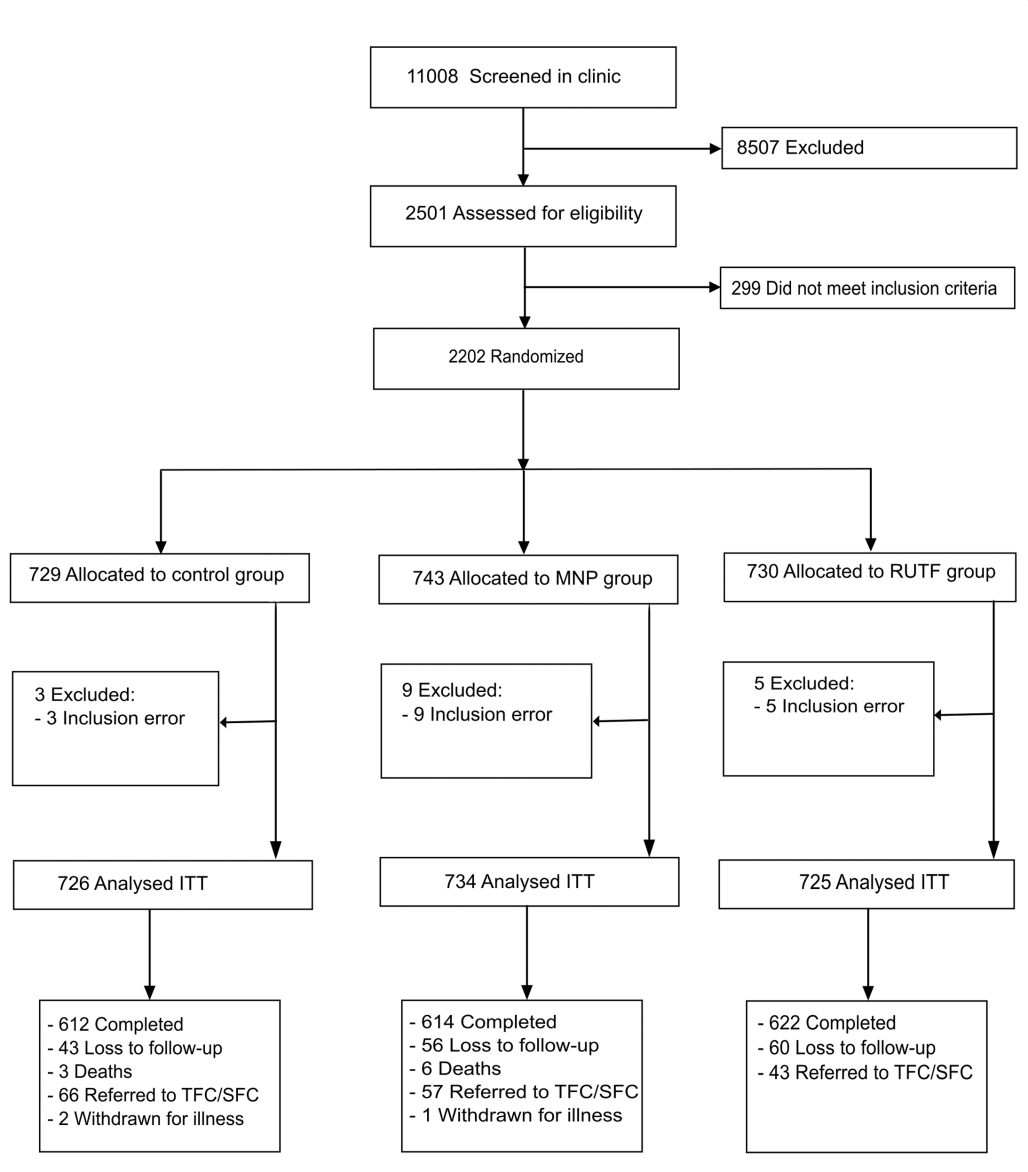
Flow diagram of participants’ supplementation study in Kaabong. ITT, intention to treat; MNP, micronutrient powder; RUTF, ready-to-use therapeutic food; TFC/SFC, therapeutic/supplementary feeding centre.

Recruitment took place from 3rd March 2011 until 4th April 2012. The last follow-up was completed on 18th September 2012. 11,008 patients younger than 5 y visiting the outpatient clinic were screened on age illness and living area; of these 2.6% were also excluded because of severe malnutrition and 8.3% for moderate malnutrition. A total of 2,202 children, aged between 6 and 59 mo, were included; 17 (0.8%) participants were excluded from analysis because of inclusion error, and 148 (6.7%) participants were lost to follow-up (abandoned the study or admitted to hospital) (Fig 1). The average number of days participants were in the study were 161.4, 161.2, and 162.9 d for RUTF, MNP, and control group, respectively.

### Baseline Characteristics

Most caretakers (93.4%) were married, and 80.4% had no formal schooling. One-quarter (24.3%) of the heads of households made a living in agriculture or livestock of their own resources. More than half of the heads of households (51.7%) were dependent on daily or seasonal labour. Only 26.4% of the households owned livestock; 7.2% owned cows, which is a sign of wealth; 9.8% of the households owned a bicycle; 8.2% had three or more meals the day before enrollment (Table 2).

**Table 2:** Baseline characteristics.

Characteristic	Value	RUTF (*n* = 725)	MNP (*n* = 734)	Control (*n* = 726)	Total (*n* = 2,185)
**Baseline household and caretakers**					
Age caretaker (years, mean ± SD)		29.2 ± 7.9	29.2 ± 8.1	29.0 ± 8.3	29.1 ± 8.1
Number of people in house (mean ± SD)	Children <5 y	2.0 ± 0.9	2.0 ± 0.9	2.1 ± 0.9	2.0 ± 0.9
	Children 5–17 y	2.4 ± 1.9	2.4 ± 1.7	2.1 ± 1.7	2.3 ± 1.8
	Adults ≥	3.4 ± 1.6	3.4 ± 1.5	3.4 ± 1.7	3.4 ± 1.6
Education of caretaker (*n* [%])	No formal school	585 (80.7%)	597 (81.7%)	575 (79.3%)	1,757 (80.4%)
	Primary school ((in-)complete)	69 (9.5%)	77 (10.5%)	71 (9.8)	217 (9.9%)
	More than primary school	70 (9.5%)	57 (7.8%)	78 (10.8%)	205 (9.4%)
	Other/unknown	1 (0.1%)	0	1 (0.1%)	2 (0.1%)
Occupation of head of household (*n* [%])	Agriculture/livestock	170 (24.6%)	189 (26.9%)	172 (25.2%)	531 (24.3%)
	Business/shop owner	13 (1.9%)	16 (2.3%)	12 (1.8%)	41 (1.9%)
	Regular paid work	89 (12.9%)	91 (13.0%)	86 (12.6%)	266 (12.2%)
	Daily/seasonal labour	386 (55.9%)	375 (53.4%)	368 (54.0%)	1,129 (51.7%)
	Housewife/other/unknown	33 (4.8%)	31 (4.4%)	43 (6.2%)	107 (5.3%)
Occupation of caretaker (*n* [%])	Agriculture/livestock	182 (25.2%)	172 (23.5%)	192 (26.3%)	546 (25.0%)
	Business/shop owner	7 (0.97%)	13 (1.8%)	14 (1.9%)	34 (1.6%)
	Regular paid work	33 (4.6%)	19 (2.6%)	20 (2.8%)	72 (3.3%)
	Daily/seasonal labour	146 (20.3%)	161 (22.0%)	139 (19.2%)	446 (20.5%)
	Housewife/other/unknown	353 (49.0%)	366 (50.0%)	361 (49.7%)	1,080 (49.6%)
Owns livestock (*n* [%])	No	546 (75.3%)	530 (72.2%)	533 (73.4%)	1,609 (73.6%)
	Yes	179 (24.7%)	204 (27.8%)	193 (26.6%)	576 (26.4%)
Owns cows (*n* [%])	No	668 (92.4%)	682 (93.2%)	675 (93.0%)	2,025 (92.9%)
	Yes	55 (7.6%)	50 (6.8%)	51 (7.0%)	156 (7.2%)
Has at least one item (*n* [%])	Watch	64 (8.9%)	70 (9.6%)	60 (8.3%)	194 (8.9%)
	Bicycle	71 (9.8%)	71 (9.7%)	72 (9.9%)	214 (9.8%)
	Radio	125 (17.3%)	135 (18.4%)	110 (15.2%)	370 (17.0%)
	Generator	6 (0.8%)	5 (0.7%)	8 (1.1%)	19 (0.9%)
Food aid (ration) (*n* [%])	No	525 (72.41%)	523 (71.3%)	527 (72.6%)	1575 (72.1%)
	Yes	200 (27.6%)	210 (28.6%)	199 (27.4%)	609 (27.9%)
	Don’t know	0	1 (0.1%)	0	1 (0.1%)
Number of meals per day (*n* [%])	0–2	653 (90.1%)	674 (91.8%)	679 (93.5%)	2,006 (91.8%)
	≥3	72 (9.9%)	60 (8.2%)	47 (6.5%)	179 (8.2%)
**Baseline children**					
Male sex (*n* [%])		345 (47.6%)	354 (48.3%)	368 (50.7%)	1,067 (48.8%)
Age (months, mean ± SD)		25.7 ± 14.2	25.2 ± 14.1	24.7 ± 13.2	25.2 ± 13.8
Aged <36 mo		448 (61.8%)	453 (61.7%)	476 (65.6%)	1,377 (63.0%)
Breastfeeding at enrollment (n [%])	None	370 (51.2%)	371 (50.8%)	351 (48.4%)	1,092 (50.1%)
	Partial	353 (48.8%)	360 (49.3%)	374 (51.6%)	1087 (49.9%)
MUAC (mm, mean ± SD)		143.8 ± 10.2	143.0 ± 9.5	143.0 ± 9.9	143.3 ± 9.8
Weight (kg, mean ± SD)		10.3 ± 2.6	10.2 ± 2.6	10.0 ± 2.4	10.2 ± 2.5
Height (cm, mean ± SD)		81.4 ± 11.5	81.5 ± 11.5	80.6 ± 10.7	81.2 ± 11.3
Weight-for-age (z-score, mean ± SD)		−1.3 ± 1.0	−1.2 ± 1.1	−1.4 ± 1.1	−1.3 ± 1.1
Height-for-age (z-score, mean ± SD)		−1.5 ± 1.5	−1.3 ± 1.6	−1.5 ± 1.5	−1.5 ± 1.6
Weight-for-height (z-score, mean ± SD)		−0.6 ± 0.8	−0.7 ± 0.8	−0.7 ± 0.8	−0.7 ± 0.8
Study disease malaria (*n* [%])^1^		218 (30.1%)	235 (32.0%)	199 (27.4%)	652 (29.8%)
Study disease LRTI (*n* [%])^1^		228 (31.5%)	262 (35.7%)	263 (36.2%)	753 (34.5%)
Study disease diarrhoea (*n* [%])^1^ ^,^ ^2^		464 (64.0%)	458 (62.4%)	457 (63.0)	1,379 (63.1%)
Days ill prior to visit (days, mean ± SD)		2.5 ± 1.4	2.4 ± 1.0	2.5 ± 1.0	2.5 ± 1.2
Recruitment period	Mar–Aug 2011	344 (47.5%)	336 (45.8%)	365 (50.3%)	1,045 (47.8%)
	Sep–Oct 2011	140 (19.3%)	135 (18.4%)	133 (18.3%)	408 (18.7%)
	Nov 2011–Apr 2012	241 (33.2%)	263 (35.8%)	228 (31.4%)	732 (33.5%)

LRTI: lower respiratory tract infection; MNP: micronutrient powder; MUAC: mid-upper arm circumference; RUTF: ready-to use therapeutic food.

^**1**^No hierarchy in study diseases.

^2^Nonbloody or bloody diarrhoea.

Most (63.0%) participants were aged 6–36 mo, and about half (49.9%) were partially breastfed. Most (63.1%) of the participants had diarrhoea on enrollment, 29.8% had malaria, and 34.5% had LRTI (more than one disease could be reported). After the onset of illness, caretakers waited for an average of 2.5 d before seeking help at the clinic. Almost half (47.8%) of the participants were enrolled in March–August 2011 and were participating in the study partly during the rainy season; 33.5% enrolled in November 2011–April 2012, and they were also partly participating in the rainy season; 18.7% were enrolled in September–October 2011 and were never participating during the rainy season. There was no clear difference between the supplementation groups in regards to these baseline characteristics (Table 2).

### Incidence of Malnutrition

The incidence of first NNO event was 0.143, 0.185, and 0.213 events per year for the RUTF, MNP, and control groups, respectively, during the 6 mo of follow-up (Table 3). The RUTF group showed a significant reduction in the incidence of NNO of 0.070 events/year or a reduction of 33.3% (*p* = 0.037) compared with the control group. The RUTF group showed a nonsignificant reduction of 0.042 events/y (22.7%) in incidence compared with the MNP group (*p* = 0.200), and the reduction in incidence in the MNP group compared with the control group was 0.028 events/y (13.8%) (*p* = 0.413).

**Table 3:** Incidence of first NNO per y.

	Incidence rate (events/y) (95% CI)			Incidence rate ratio (95% CI)			*p*-value		
	RUTF	MNP	Control	RUTF versus MNP	RUTF versus control	MNP versus control	RUTF versus MNP	RUTF versus control	MNP versus control
ITT	0.143 (0.107;0.191)	0.185 (0.141;0.239)	0.213 (0.167;0.272)	0.773 (0.522;1.146)	0.667 (0.455;0.976)	0.862 (0.605;1.229)	0.200	0.037	0.413
PP	0.122 (0.085;0.174)	0.152 (0.106;0.215)	0.198 (0.150;0.261)	0.804 (0.487;1.328)	0.617 (0.393;0.971)	0.768 (0.491;1.202)	0.394	0.037	0.248

ITT: intention to treat; MNP: micronutrient powder; NNO: negative nutritional outcome; PP: per protocol; RUTF: ready-to-use therapeutic food; Number analysed: ITT *n* = 2,171; PP *n* = 1,558

Analysis of the per protocol dataset (*n* = 1,558) did not show a major difference from the intention to treat dataset. The incidence of NNO was 0.122 events/y for the RUTF group, 0.152 events/y for the MNP group, and 0.198 events/y for the control group during the 6 mo of follow-up. The incidence in the RUTF group was 39.3% lower compared with the control group (*p* = 0.037) and 19.6% lower compared with the MNP group (*p* = 0.394), and the incidence in the MNP group was 23.8% lower than the control group (*p* = 0.248) (Table 3).

A total of 166 participants developed moderate malnutrition (weight-for-height z-score between −2 and −3): 44 (6.09%) in the RUTF group, 56 (7.70%) in the MNP group, and 66 (9.14%) in the control group. Sixteen participants developed severe malnutrition (weight-for-height z-score <−3, MUAC <115 mm, or oedema): 4 (0.55%) in the RUTF group, 9 (1.24%) in the MNP group, and 3 (0.42%) in the control group. None of the participants developed oedema.

Subgroup analyses suggested that the effect of supplementation on the incidence of NNO was not modified by socioeconomic characteristics, season of enrollment, age of the participant, breastfeeding status, or study disease at enrollment (Table 4).

**Table 4:** Incidence rate of first NNO per y by subgroups.

Subgroup		*n*	*p-Value for interaction* ^1^	NNO incidence rate (95% CI)			Incidence rate ratio (95% CI)		
				RUTF	MNP	Control	RUTF versus MNP	RUTF versus control	MNP versus control
**Caretaker/household**									
Owns livestock	Yes	571	0.149	0.117 (0.061;0.224)	0.224(0.143;0.352)	0.143 (0.0807;0.252)	0.518 (0.234;1.145)	0.815 (0.344;1.935)	1.574 (0.764;3.243)
	No	1,600		0.1520 (0.109;0.211)	0.169 (0.124;0.232)	0.241 (0.185;0.313)	0.894 (0.565;1.415)	0.629 (0.411;0.963)	0.704 (0.465;1.066)
Owns cows	Yes	155	0.849	0.176 (0.065;0.467)	0.189 (0.070;0.502)	0.178 (0.067;0.474)	0.934 (0.234;3.734)	0.985 (0.246;3.940)	1.055 (0.264;4.220)
	No	2,012		0141 (0.102;0.191)	0.180 (0.137;0.237)	0.217 (0.169;0.278)	0.776 (0.514;1.171)	0.648 (0.435;0.964)	0.835 (0.578;1.208)
Education	Low	1,748	0.895	0.148 (0.106;0.202)	0.176 (0.133;0.237)	0.224 (0.172;0.291)	0.830 (0.537;1.283)	0.656 (0.433;0.996)	0.790 (0.533;1.172)
	Middle	217		0.100 (0.033;0.313)	0.217 (0.104;0.458)	0.161 (0.067;0.387)	0.462 (0.119;1.787)	0.628 (0.150;2.627)	1.359 (0.431;4.281)
	High	200		0.148 (0.056;0.393)	0.183 (0.070;0.487)	0.191 (0.087;0.426)	0.810 (0.203;3.239)	0.774 (0.219;2.744)	0.956 (0.270;3.388)
**Child**									
Age	<36 mo	1,365	0.090	0.150 (0.102;0.215)	0.219 (0.163;0.300)	0.280 (0.215;0.363)	0.677 (0.419;1.095)	0.532 (0.338;0.837)	0.786 (0.525;1.176)
	≥36 mo	806		0.133 (0.080;0.215)	0.130 (0.080;0.213)	0.091 (0.050;0.169)	1.017 (0.509;2.034)	1.440 (0.654;3.174)	1.416 (0.643;3.120)
Sex	Male	1,060	0.589	0.137 (0.187;0.211)	0.215 (0.152;0.304)	0.230 (0.167;0.319)	0.631 (0.361;1.103)	0.591 (0.342;1.021)	0.937 (0.582;1.508)
	Female	1,111		0.148 (0.100;0.222)	0.154 (0.104;0.230)	0.196 (0.137;0.280)	0.954 (0.545;1.671)	0.754 (0.441;1.289)	0.790 (0.464;1.343)
Breastfeeding	No	1,084	0.137	0.137 (0.091;0.209)	0.117 (0074;0.185)	0.133 (0.085;0.204)	1.169 (0.633;2.160)	1.039 (0.567;1.903)	0.889 (0.474;1.665)
	Partial	1,081		0.148 (0.098;0.226)	0.259 (0.189;0.356)	0.293 (0.219;0.391)	0.572 (0.338;0.967)	0.508 (0.306;0.844)	0.888 (0.578;1.365)
Recruitment period	Mar–Aug 2011	1,034	0.138	0.146 (0.096;0.224)	0.241 (0.172;0.339)	0.269 (0.198;0.365)	0.605 (0.350;1.045)	0.543 (0.321;0.919)	0.898 (0.568;1.419)
	Sep–Oct 2011	405		0.135 (0.067;0.272)	0.193 (0.106;0.348)	0.087 (0.035;0.206)	0.705 (0.283;1.752)	1.580 (0.517;4.828)	2.241 (0.779;6.451)
	Nov 2011–Apr 2012	732		0.141 (0.085;0.235)	0.113 (0.065;0.193)	0.204 (0.130;0.315)	1.256 (0.598;2.640)	0.694 (0.355;1.356)	0.553 (0.275;1.111)
Study disease	Only diarrhoea^2^	941	0.391	0.141 (0.089;0.222)	0.209 (0.143;0.306)	0.228 (0.161;0.324)	0.675 (0.374;1.220)	0.618 (0.349;1.095)	0.916 (0.544;1.542)
	Only LRTI	341		0.063 (0.022;0.198)	0.206 (0.111;0.385)	0.185 (0.100;0.345)	0.310 (0.085;1.125)	0.345 (0.095;1.254)	1.114 (0.464;2.677)
	Only malaria	316		0.096 (0.039;0.228)	0.126 (0.056;0.282)	0.074 (0.024;0.228)	0.750 (0.229;2.457)	1.296 (0.310;5.421)	1.728 (0.432;6.908)
	≥2 diseases	573		0.226 (0.141;0.365)	0.167 (0.102;0.278)	0.280 (0.185;0.426)	1.352 (0.675;2.706)	0.808 (0.429;1.522)	0.598 (0.310;1.153)
Days of illness before enrollment	1 or 2	1,395	0.448	0.117 (0.078;0.174)	0.183 (0.130;0.252)	0.200 (0.146;0.274)	0.644 (0.384;1.080)	0.584 (0.351;0.971)	0.906 (0.576;1.426)
	3	565		0.217 (0.133;0.356)	0.163 (0.096;0.276)	0.248 (0.161;0.385)	1.337 (0.653;2.740)	0.877 (0.454;1.692)	0.656 (0.331;1.298)
	≥4	184		0.113 (0.037;0.350)	0.248 (0.102;0.593)	0.156 (0.065;0.378)	0.456 (0.109;1.909)	0.718 (0.171;3.003)	1.573 (0.455;5.432)

LRTI: lower respiratory tract infection; MNP: micronutrient powder; RUTF: ready-to-use therapeutic food.

^1^ *p* for interaction between subgroups on differences between arms.

^2^Nonbloody or bloody diarrhoea.

This is despite the fact that in the control group, some subgroups (e.g., partially breastfed, children younger than 36 months, those enrolled in the wet seasons) did appear to be at higher risk of developing a NNO. (Table 4).

Whereas MNP did not show a significant effect on the incidence of NNO, a positive effect of MNP was seen for the changes in individual anthropometric indicators of participants who completed the study (Table 5).

**Table 5:** Anthropometric indicators: change from baseline to day 14 and day 168.

Indicator		Mean/proportion (95% CI)			Mean difference/odds ratio (95% CI)			*p*-Value		
	*n*	RUTF	MNP	Control	RUTF versus MNP	RUTF versus control	MNP versus control	RUTF versus MNP	RUTF versus control	MNP versus control
**Day 0–day 14**										
Mean rate weight gain (g/kg/d)^1^	1,877	1.64 (1.46;1.83)	1.57 (1.38;1.76)	1.25 (1.06;1.44)	0.07 (−0.19;0.34)	0.40 (0.13;0.66)	0.32 (0.05;0.59)	0.588	0.004	0.018
Not gaining weight (%)	1,877	31% (22%;42%)	35% (25%;46%)	43% (32%;54%)	0.86 (0.66;1.11)	0.61 (0.47;0.78)	0.71 (0.55;0.91)	0.246	0.0001	0.006
Mean MUAC^4^ gain (mm/d)^2^	1,877	0.062 (0.044;0.080)	0.040 (0.220;0.057)	0.032 (0.014;0.051)	0.023 (−0.003;0.048)	0.03 (0.004;0.055)	0.007 (−0.019;0.033)	0.082	0.024	0.586
**Day 0–day 168**										
Rate weight gain (g/kg/d)^1^	1,997	0.64 (0.61;0.66)	0.65 (0.62;0.67)	0.60 (0.57;0.62)	−0.01 (−0.05;0.03)	0.04 (0.00;0.08)	0.05 (0.01;0.09)	0.590	0.031	0.007
MUAC change rate (mm/day)^2^	1,997	0.014 (0.012;0.017)	0.014 (0.012;0.017)	0.009 (0.006;0.011)	−0.00 (−0.004;0.004)	0.006 (0.002;0.009)	0.006 (0.002;0.010)	0.894	0.003	0.002
Height change (cm)^3^	1,995	3.02 (2.92;3.11)	3.09 (3.00;3.19)	3.01 (2.92;3.10)	−0.08 (−0.21;0.05)	0.01 (−0.12;0.14)	0.08 (−0.05;0.21)	0.239	0.934	0.206
Weight/Age change (z-scores)^1^	1,996	0.04 (0.00;0.08)	0.04 (0.00;0.08)	−0.03 (−0.07;0.01)	−0.00 (−0.06;0.05)	0.07 (0.02;0.12)	0.07 (0.02;0.13)	0.914	0.012	0.009
Height/Age change (z-scores)^3^	1,994	−0.51 (−0.57;−0.46)	−0.51 (−0.57;−0.46)	−0.55 (−0.60;−0.49)	0.00 (−0.07;0.08)	0.03 (−0.04;0.11)	0.03 (−0.04;0.11)	0.970	0.374	0.393
Weight/Height change (z-scores) ^3^	1,994	0.41 (0.36;0.46)	0.40 (0.35;0.45)	0.33 (0.28;0.38)	0.01 (−0.06;0.08)	0.07 (0.00;0.14)	0.06 (−0.01;0.14)	0.815	0.043	0.073

MNP: micronutrient powder; MUAC: mid-upper arm circumference; RUTF: ready-to-use-therapeutic food.

^1^Adjusted for weight at baseline.

^2^Adjusted for MUAC at baseline.

^3^Adjusted for height at baseline.

The weight gain rate at day 168 was significantly higher for the supplementation groups compared with the control group: 0.64, 0.65, and 0.60 g/kg/d for RUTF, MNP, and control groups, respectively (RUTF versus control, *p* = 0.031; MNP versus control, *p* = 0.007). The MUAC gain rate at day 168 was also higher for the supplementation groups compared with the control group: 0.014, 0.014, and 0.009 mm/d for RUTF, MNP, and control groups, respectively (RUTF versus control, *p* = 0.003; MNP versus control, *p* = 0.002) (Table 5).

Similarly, the change in weight-for-age index was higher for the supplementation groups than for the control group: z-scores of 0.04, 0.04, and −0.03 for RUTF, MNP, and control groups, respectively (RUTF versus control, *p* = 0.012; MNP versus control, *p* = 0.009). Finally, the change in weight-for-height index was also higher for the supplementation groups than the control group: z-scores of 0.41, 0.40, and 0.33 for RUTF, MNP, and control groups, respectively (RUTF versus control, *p* = 0.043; MNP versus control, *p* = 0.073). The change in height and the change in height-for-age index were not different between the supplementation arms (Table 5).

In the first 14 d, directly after the first supplementation, the RUTF and MNP groups showed a significantly higher weight gain rate compared with the control group: 1.64, 1.57, and 1.25 g/kg/d for RUTF, MNP, and control groups, respectively (RUTF versus control, *p* = 0.004; MNP versus control, *p* = 0.018). Similarly, a lower proportion of participants did not gain weight in the supplementation groups. The proportion of children not gaining weight was 31%, 35%, and 43% for RUTF, MNP, and control groups, respectively (RUTF versus control, *p* = 0.0001; MNP versus control, *p* = 0.006) (Table 5).

### Disease and Mortality

The proportion of children experiencing one or more new episodes of malaria during the follow-up was 47%, 43%, and 51% for the RUTF, MNP, and control groups, respectively (Table 6). The MNP group showed a lower proportion of children having a new malaria episode compared with the control group (*p* = 0.001). The proportion of children experiencing one or more new diarrhoea episodes was 44%, 44%, and 46% in the RUTF, MNP, and control groups, respectively; for LRTI, the proportions were 43%, 44%, and 46%. The average number of malaria episodes was 0.79, 0.70, and 0.80 for the RUTF, MNP, and control groups, respectively (MNP versus control, *p* = 0.075); the average number of diarrhoea episodes was 0.78, 0.73, and 0.80, and the average number of LRTI episodes was 0.71, 0.68, and 0.72. The mean number of all diagnosed study diseases was 2.29, 2.12, and 2.31, for the RUTF, MNP, and control groups, respectively (Table 6).

**Table 6:** Mean number of diagnosed events and proportion of children with at least one newly diagnosed study disease episode^1^.

Disease	Mean/Proportion (95% CI)			Mean difference/odds ratio (95% CI)			*p*-Value		
	RUTF	MNP	Control	RUTF versus MNP	RUTF versus control	MNP versus control	RUTF versus MNP	RUTF versus control	MNP versus control
	**Mean (95% CI) number of diagnosed study disease (95%CI)**			**Mean difference (95% CI)**					
Malaria,	0.79 (0.72;0.86)	0.70 (0.63;0.77)	0.80 (0.72;0.87)	0.09 (−0.01;0.19)	−0.01 (−0.11;0.10)	−0.09 (−0.20;0.01)	0.094	0.916	0.075
LRTI	0.71 (0.64;0.79)	0.68 (0.61;0.76)	0.72 (0.64;0.79)	0.03 (−0.07;0.13)	−0.00 (−0.11;0.10)	−0.04 (−0.14;0.07)	0.558	0.938	0.506
Diarrhoea^2^	0.78 (0.70;0.87)	0.73 (0.65;0.81)	0.80 (0.72;0.88)	0.05 (−0.06;0.17)	−0.01 (−0.13;0.10)	−0.06 (−0.18;0.05)	0.386	0.813	0.270
All 3 diseases	2.29 (2.15;2.42)	2.12 (1.98;2.25)	2.31 (2.17;2.45)	0.17 (−0.03;0.36)	−0.02 (−0.22;0.17)	−0.19 (−0.39;0.00)	0.088	0.813	0.052
	**Proportion (95% CI) of children having ≥1 new disease**			**Odds ratio (95% CI)**					
Malaria	47% (43;50)	43% (39;46)	51% (47;54)	1.18 (0.96;1.46)	0.85 (0.69;1.04)	0.72 (0.58;0.88)	0.110	0.114	0.001
LRTI	43% (39;46)	44% (40;47)	46% (42;49)	0.96 (0.78;1.18)	0.89 (0.72;1.09)	0.93 (0.75;1.14)	0.678	0.266	0.484
Diarrhoea^2^	44% (40;47)	44% (40;47)	46% (42;49)	1.00 (0.81;1.23)	0.92 (0.75;1.14)	0.93 (0.75;1.14)	0.965	0.459	0.484

LRTI: lower respiratory tract infection; MNP: micronutrient powder; RUTF: ready-to-use therapeutic food.

^1^ *n* = 2,171 (excluding 14 with no follow-up data).

^2^Nonbloody or bloody diarrhoea.

A total of nine children died during the study duration: none in the RUTF group, six in the MNP group, and three in the control group. Among the nine deaths, three occurred at the hospital (Table 7). The causes of deaths in the hospital were severe malaria and severe anaemia in two patients and bloody diarrhoea in one patient (all in the control group). The other six patients died at home, and the caretakers did not know the cause of death. A total of 14 participants were admitted to the hospital (not deceased): six in the RUTF group, two in the MNP group, and six in the control group. In total, 23 participants were admitted to the hospital or died: six in the RUTF group, eight in the MNP group, and nine in the control group (Table 7).

**Table 7:** Overall mortality and hospital admission by intervention group

Event	Category	RUTF (*n* = 725)		MNP (*n* = 734)		Control (*n* = 726)		Total (*n* = 2,185)	
		*n*	%	*n*	%	*n*	%	*n*	%
**Deaths** ^**1**^									
Total deaths		0^1^	0%	6^1^	0.8%	3^1^	0.4%	9	0.4%
Cause	Malaria	0	0%	0	0%	2	0.3%	2	0.1%
	LRTI	0	0%	0	0%	0	5%	0	0
	Diarrhoea/dehydration	0	0%	0	0%	1	0.1%	1	0%
	Unknown	0	0%	6	0.8%	0	0%	6	0.3%
**Hospital admissions**									
Total hospital admissions		6	0.8%	2	0.3%	6	0.8%	14	0.6%
Cause	Malaria	4	0.6%	2	0.3%	1	0.1%	7	0.3%
	LRTI	1	0.1%	0	0%	0	0%	1	0%
	Diarrhoea/dehydration	0	0%	0	0%	2	0.3%	2	0.1%
	Other^2^	1	0.1%	0	0%	3	0.4%	4	0.2%

LRTI: lower respiratory tract infection; MNP: micronutrient powder; RUTF: ready-to-use therapeutic food.

^1^RUTF significantly lower mortality versus MNP. Fischer’s exact test *p*: RUTF versus MNP, 0.031; RUTF versus control, 0.250; MNP versus control, 0.341.

^2^Nephrotic syndrome, burns, unknown (6), head trauma.

### Consumption and Compliance

Caregivers of children taking RUTF or MNP were asked about the consumption of sachets during the 2 wk following allocation. Reported consumption over the first 14-d period directly after enrollment showed that participants in the RUTF group consumed on average 13.7 (out of 14) sachets, and participants in the MNP group consumed on average 24.3 (out of 28) sachets (Table 8). Reported consumption over the entire study period and all allocations showed an average of 13.8 (out of 14) sachets consumed per participant per allocation in the RUTF group and 24.7 (out of 28) sachets consumed per participant per allocation in the MNP group.

**Table 8:** Allocation of and compliance with supplements in intervention group.

Measure	RUTF		MNP		Control	
	Day 0–14	Study period	Day 0–14	Study period	Day 0–14	Study period
Number of allocations assigned	730	1,629	743	1,599	729	1,642
Number of consumption reports (*n*, % of allocations)	500 (68.5%)	984 (60.4%)	426 (57.3%)	895 (57.4%)		
Number of sachets consumed (mean ± SD)	13.7 (±1.4)	13.8 (±1.0)	24.3 (±6.6)	24.7 (±5.9)		
Consumed 14 for RUTF or 28 for MNP (*n*, % of allocations))	470 (94.0%)	940 (95.5%)	294 (6.0%)	667 (74.5%)		

MNP: micronutrient powder; RUTF: ready-to-use therapeutic food.

Focus group discussions showed that all mothers enjoyed giving RUTF or MNP to their children. About RUTF, they said the "children gained weight and improved faster than they usually did.” The children liked the RUTF so much that some even tried “grabbing it” from the mothers. All caregivers reported that they did not give any of the supplements to a nonstudy child because it had been prescribed as “medicine” for the sick child. They all denied having sold or shared any of the supplements. All the caregivers said that it was easy to use the MNP and they felt that the supplement was very important for the child to recover from their illness.

## Discussion

### Incidence of Malnutrition

This study and its companion trial in Goronyo (Nigeria) are the first large randomised controlled trials of the use of high quality supplements (RUTF and MNP) for the prevention of malnutrition in children with a nonsevere illness. In our site in Kaabong, Uganda, we showed that 14 d of supplementing ill children with RUTF was effective in lowering the incidence of malnutrition. The RUTF supplementation group showed a significant reduction in the incidence of NNO of 33% compared with the control group. Supplementation with MNP showed a nonsignificant reduction of 13.8%. Both RUTF and MNP supplementation had a significant positive effect on anthropometric indicators such as rate of weight gain and gain in MUAC. There were fewer deaths observed in the RUTF group than in the control and MNP groups, but the numbers were too low to draw clear conclusions.

In contrast, in the companion study in Nigeria, there was no reduction in incidence of malnutrition with short-term supplementation of either RUTF or MNP compared with a control group, and there was no impact on any of the anthropometric indices. It is postulated that the high morbidity in Nigeria necessitates a higher dose or a longer duration of supplementation for effectiveness. In addition, the supplements were not effective among the moderately malnourished children included in the study in Nigeria. Children with moderate acute malnutrition (MAM), while at higher risk of progressing to severe acute malnutrition (SAM), generally require 4 wk or more of treatment to fully recover their nutritional status so it may be that the 14 d period of supplementation was insufficient. Similar to our findings in this study, there was a trend to fewer deaths in the RUTF group compared with the control or MNP group in the Nigerian study [29].

The findings in Kaabong support a study on the effect of supplementing all children (not necessarily ill) in Niger that also showed a reduction in malnutrition. A supplementation of one sachet of RUTF daily for a period of 3 mo during the hunger season in children led to a significantly lower incidence of malnutrition (weight-for-height z-scores) in the supplemented group after a follow-up period of 8 mo. The incidences in the RUTF and control groups were, respectively, 0.17 and 0.26 per child per year; a reduction in incidence of malnutrition of 35% in the RUTF group [35].

The overall incidence of malnutrition among ill children included in our study was remarkably low in Kaabong. The incidence of a first event of malnutrition in the control group was 0.21 events/y, whereas we had anticipated an incidence of first time events of 0.44 events/y in the control group. In comparison, in the companion study in Nigeria, the incidence in the control group was 0.57 events/y and 0.59 events/y when excluding moderate malnourished [29]. A study among sick and nonsick children in Niger during the hunger gap also showed an incidence of 0.26 events/y [35].

The lower incidence of malnutrition in Kaabong in our study participants could be due to the changed context of a reduced intensity of the conflict and improved security. At the time the study was being planned, security in Karamoja was hampered owing to clashes between tribes; when the study was implemented, the region had stabilised.

The difference in incidence rate between the RUTF and control groups over the observation period of 6 mo was 0.032; the number needed to treat (NNT) was 31. This implies that 31 non-malnourished, ill children need to be supplemented after an illness during 6 mo to prevent 1 case of NNO in this group. In the situation of Kaabong, with a relatively low incidence of malnutrition, it is more efficient to target specific groups within the group of ill children with a higher incidence of malnutrition (but a similar Incidence Rate Ratio [IRR]), to reduce the NNT—for example, partially breastfed children younger than 3 y who have an overall higher incidence of malnutrition, or supplementation during the rainy season.

The MNP supplementation did not show a significant effect on the incidence of malnutrition among ill children. However, both RUTF and MNP seem to have a significant effect on several anthropometric indicators: higher mean weight gain rate, MUAC gain, weight-for-height gain, and weight-for-age gain at the end of the study. A very direct effect was shown by the weight gain at day 14 immediately after supplementation. Both MNP and RUTF groups showed a higher weight gain and also a lower percentage of participants who did not gain weight at all after 14 d of follow-up. These data show that both RUTF and MNP improved the nutritional status, such as weight and MUAC gain, but that RUTF was more effective in preventing children falling below the threshold of malnutrition. A general improvement in nutritional status is beneficial; however, from a clinical point of view, reduction in the incidence of malnutrition is crucial to reduce mortality among under-5s.

The promising results with micronutrients alone (MNP), and the clear effect of a high quality food with 500 kcal (RUTF), suggest that a supplement with a lower energy content might also be effective in reducing malnutrition. After this study was designed, more types of fortified foods became available—these are generally called lipid-based supplements and contain varying quantities of fat, carbohydrates, and proteins and a varying micronutrient composition. Compared with RUTF, these foods are more adapted to the needs of very young non-malnourished or moderately acutely malnourished children. In a study in Burkina Faso, children aged 9 mo (from the general population) receiving a low quantity lipid-based supplement and relevant medical care for 12 mo showed a significant reduction in wasting [36]. However, a study in Chad using the same supplement for 4 mo in children from the general population alongside general food distribution failed to show any effect of the supplementation in children aged 6–36 mo [37]. Unlike our study and the study in Burkina Faso, no specific morbidity surveillance and care was given in Chad, which might be a crucial element in preventing malnutrition for these age groups. Thus, from a cost-effectiveness point of view, it seems important to investigate whether supplementation with products containing less energy (lipid-based supplements, medium quantity) provided alongside morbidity surveillance and treatment is also effective in reducing the incidence of acute malnutrition in ill children.

### Morbidity and Mortality

The effect of supplementation on further morbidity in ill children was mixed. The average number of study diseases diagnosed was 2.29, 2.12, and 2.31 study diseases for RUTF, MNP, and control groups, respectively. The proportion of children having a newly diagnosed episode of diarrhoea or LRTI during the study was similar among the study groups. However, the MNP group had a lower average number of malaria episodes than the control group, as well as a lower proportion of children having at least one new episode of malaria. These findings are curious, as a malaria infection is unlikely to be influenced by supplementation. Possibly, the clinical expression is slightly different (for example, less fever), which might influence the decision to perform a rapid malaria test and thus result in a lower incidence of malaria episodes in the MNP group.

Only 14 children required admission to hospital (of which nine were due to malaria), and the MNP group had the lowest number of hospital admissions. However, the RUTF group had no mortality; three children died in the control group, and the MNP group showed the highest mortality with six cases. The mortality data are difficult to interpret because of the low numbers and lack of data on the cause of death for the participants who died at home. It is unlikely that the study underestimated mortality, as all participants who stopped attending the study were followed up, and information from the family and their neighbours was obtained on the whereabouts of the child. When taken together, the RUTF group showed a tendency to have a lower risk of hospital admission and death. A similar trend was noted in the companion study in Goronyo.

### Risks

A study in Pakistan found an increased risk of severe diarrhoea and severe LRTI in the MNP group [38], but this was not confirmed by our study.

Some literature suggests that supplements containing iron might worsen the outcome of malaria episodes [39,40]. Systematic reviews did not confirm this concern [41,42]. In our study, we used two doses of micronutrients resulting in twice the amount of recommended iron of 20 mg instead of 10 mg per day. Although the number of deaths were highest in the MNP group (6 versus 0 in the RUTF and 3 in the control group), the causes were unknown. On the other hand, the MNP group had the lowest number of the hospitalized patients related to malaria. These data neither confirm nor reject the concern that malaria has a more severe outcome in the MNP group.

The data does not show any clear negative effect of the supplement on severe morbidity or mortality. It also does not confirm concerns that supplements might increase the risk on severe outcome related to diarrhoea or malaria.

Finally, the compliance and focus group results demonstrated that RUTF and MNP are both acceptable in terms of correct use, likeability, organoleptic properties (such as colour and taste), side effects, and compliance.

### Limitations

Despite the robust study protocols and strict implementation, this study has some limitations. First of all, the participants were not blinded for the RUTF or MNP supplements. As the population had possibly a prior positive attitude towards RUTF, this could introduce a bias to more positive reporting and compliance compared with MNP. However, blinding is difficult to achieve, and our efforts to produce a placebo for MNP failed. Nevertheless, because MNP was new to the population, we did not anticipate any positive or negative feelings about the product.

Because of the presence of feeding programmes of other organisations for treatment of severe and moderate malnourished children, we did not enroll moderate or severe malnourished children in the research but referred them instead. Therefore, the results cannot be generalised to the overall group of ill children presenting at a clinic, as these usually include acutely malnourished children. Caution should therefore be used in comparing the results with other trials, such as the study in Goronyo that did include moderate malnourished children. Also, the results cannot be extrapolated to the general population of all children (ill and not ill), and comparison with research implemented in the general population of children should be approached with caution.

Finally, although the number of lost-to-follow-up was lower than anticipated, this still might have influenced the outcome. As their outcome is unknown, some may have been malnourished and may represent missed endpoints, giving an underestimation of NNO.

### Conclusions

A 2-wk nutrition supplementation programme with RUTF as part of routine primary medical care to non-malnourished children with malaria, LRTI, or diarrhoea was effective in preventing malnutrition. The low incidence of malnutrition in this population may warrant a more targeted intervention to improve cost-effectiveness, such as ill children younger than 3 y or during the hunger season.

While supplementation with MNP showed a positive trend for several nutritional indicators, it seems that a certain level of macronutrients is needed to prevent malnutrition. Therefore, further research should focus on an appropriate balance between macro- and micronutrients to optimise the cost effectiveness of supplementation with lipid-based fortified foods in preventing malnutrition in ill children.

## Supporting Information

S1 Data Line list endpoints. (TXT)

S1 Text Protocol. (PDF)

S2 Text SAP. (PDF)

S3 Text CONSORT Checklist. (PDF)

## Abbreviations

IRR: Incidence Rate Ratio
LRTI: lower respiratory tract infection
MAM: moderate acute malnutrition
MNP: multi-micronutrient powder
MSF: Médecins Sans Frontières
MUAC: mid-upper arm circumference
NNO: negative nutritional outcome
NNT: number needed to treat
RDT: rapid diagnostic test
RUTF: ready-to-use therapeutic food
SAP: Statistical Analysis Plan
SAM: severe acute malnutrition
UNCST: Uganda National Council of Science and Technology
WHO: World Health Organization
