# Supplementary material for: Effect of Short-Term Supplementation with Ready-to-Use Therapeutic Food or Micronutrients for Children after Illness for Prevention of Malnutrition: A Randomised Controlled Trial in Uganda
Source: PLoS Med. 2016 Feb 9;13(2):e1001951. doi: 10.1371/journal.pmed.1001951 (PMC4747529; doi:10.1371/journal.pmed.1001951)
Supplement: S2 Text — (PDF) [file pmed.1001951.s003.pdf]

Effectiveness of nutritional supplementation (RUTF and multi micronutrient) in preventing malnutrition in children 6-59 months with infection (malaria, pneumonia, diarrhoea), a randomized controlled trial in Kaabong Hospital. Karamoja, Uganda

Statistical Analysis Plan

Authors: Stephanie Roll, Todd Swarthout[1], Saskia van der Kam[2].
Version: 19 July 2012

[1] Former Médecins Sans Frontières (OCA), Amsterdam, The Netherlands
[2] Co-Investigator of the present study and Principal Investigator of the Nigeria Study, Médecins Sans Frontières (OCA), Amsterdam, The Netherlands

Signature page

**Stephanie Roll**<br><br>Institute for Social Medicine, Epidemiology and Health Economics, Charité University Medical Center, Berlin, Germany	....<br>Place, Date Signature
**Núria Salse Ubach**<br><br>Médecins Sans Frontières Barcelona	....<br>Place, Date Signature

Table of contents

Signature page 2
1 Aim of the study 4
1.1 Primary objective 4
1.2 Secondary objectives 4
2 Study design 4
3 Endpoints 5
3.1 Primary endpoint 5
3.2 Secondary endpoints 5
3.3 Safety measures 5
4 Populations for analysis 5
4.1 Intention to treat population 5
4.2 Per protocol population 6
4.3 Completed the study 6
4.4 Baseline drop-out 6
5 Statistical analysis 6
5.1 Analysis of baseline data 6
5.2 Compliance 6
5.3 Primary analysis of primary endpoint 7
5.4 Secondary analysis of the primary endpoint 7
5.4.1 Treatment group comparisons 7
5.4.2 Further adjustments 8
5.4.3 Subgroups 8
5.4.4 Missing endpoint data 8
5.4.5 Per protocol analysis 8
5.5 Analysis of secondary endpoints 9
5.6 Analysis of safety measures 9
5.7 Joint analysis of data from other studies 9
5.7.1 Pooled analysis I 9
5.7.2 Pooled analysis II 9
6 Software 9

1 Aim of the study

To determine the effectiveness of 14 days nutritional supplementation (RUTF or MNP) given concurrently with the appropriate medical treatment, amongst children diagnosed with malaria and/or diarrhoea and/or LRTI in reducing the incidence of acute malnutrition and the incidence of the 3 study diseases compared to a control group.

1.1 Primary objective

The primary objective is to determine the effectiveness of 14 days of RUTF supplementation versus no supplementation (control group) on the incidence of acute malnutrition

Primary Hypothesis
Supplementation for 14 days with RUTF, concurrently with appropriate medical treatment for malaria, and/or LRTI, and/or diarrhoea reduces the incidence of acute malnutrition compared to a control group during 6 months follow-up, amongst children 6-59 months of age with malaria and/or, LRTI and/or diarrhoea at time of recruitment.

1.2 Secondary objectives

Secondary objectives include the following.
- Incidence of malnutrition

Determine the efficacy of 14 days MNP supplementation versus a control group and RUTF versus MNP on the incidence of acute malnutrition during 6 months follow-up,

- Frequency of morbidity

Determine the effect of 14 days supplementation on the frequency of morbidity (3 study diseases) during 6 months follow-up, comparing both intervention groups to the control group and comparing RUTF to MNP)

- Weight changes immediately after intervention period, measured at day-14 and day-28

Measure mean weight change (total change and rate (grams/kg/day) of change), comparing both intervention groups separately (RUTF and MNP) to the control group
Determine frequency of 'no weight gain and/or weight loss', comparing both intervention groups separately (RUTF and MNP) to the control group
Assess if provision of 14 days of RUTF or MNP supplement promotes a sustained weight gain, measured after 28-days of follow-up, comparing both intervention groups separately (RUTF and MNP) to the control group

- Evaluation of several subgroup to assess potential effect modifiers.

2 Study design

This is a partially blinded randomized controlled trial with three study groups. Children 6 to 59 months of age presenting at the OPD in Kaabong and diagnosed with one or more of the three study diseases (malaria, diarrhoea and LRTI) are eligible for the study.

Children participating in this study will be randomised to one of 3 study groups to:
A) Receive 14 days of RUTF supplementation with standard care and treatment or
B) Receive 14 days MNP supplementation with standard care and treatment or
C) Be included in a control group receiving standard care and treatment but not receiving nutritional supplementation (control group)

Individual follow-up will be 6 months. During this time, children in the RUTF or MNP group will receive 14 days nutritional supplement every time diagnosed with at least one of the three study diseases, not exceeding more than 14 days supplementation in any 28 day period.

3 Endpoints

3.1 Primary endpoint

The primary endpoint is “negative nutritional outcome (NNO)” of a child within the 6 months follow-up period. The incidence of a negative nutritional outcome will be defined in two different ways according to the baseline nutritional status:

- for children with no malnourishment at time of entry into study, “negative nutritional outcome” is defined as progression to moderate or severe malnourishment,

Thus, the event of a child reaching a “negative nutritional outcome” at least once within 6 months of follow-up period will be compared between the three study groups.

3.2 Secondary endpoints

Secondary endpoints include

- Number of NNO’s per study group in a period of 6 months
- Time to NNO
- Number of new events of a study disease in 6 months
- Weight change at 14 days and 28 days after study inclusion, and at end of study period
- Rate of weight change (grams/kg/day) at 14 days and 28 days after study inclusion, and at end of study period
- Proportion of children not gaining weight (including losing weight), measured at day-14 and day-28 after study inclusion.
- Proportion of children developing bilateral oedema, overall and per month
- Proportion of moderate acutely malnourished children improving to a non acutely malnourished state, overall and per month
- Mortality: overall mortality
- Morbidity, including the three study diseases (proportion of children with newly diagnosed diseases), overall and per month
- Proportion of children with a reported cough at clinical visit, overall and per month
- Proportion appetite categories
- Proportion and time pattern of children who were breast-fed at baseline and stopped being breast-fed during the study

3.3 Safety measures

- Serious adverse events
- Serious adverse reactions

4 Populations for analysis

4.1 Intention to treat population

The intention to treat population (ITT) includes all patients randomised that received at least one dose of study medication and who have baseline data recorded.

4.2 Per protocol population

The per protocol population (PP) includes all patients of the ITT population which were treated following the specifications of the study protocol without major protocol deviations. Excluded from the per protocol population will be patients that meet at least one of the following criteria:

- Violation of one of the following exclusion criteria: exclusive breastfeeding, hospitalisation for serious complicated illness (e.g. chronic illness).
- Not treated according to the allocated intervention group
  - For the RUTF group (for each 14-day supplementation period)
    - less than 12 days of supplement taken by the child
    - less than 12 sachets consumed by the child
    - less than 12 empty sachet counted
  - For the MNP group (for each 14-day supplementation period)
    - less than 24 days of supplement taken by the child
    - less than 24 sachets consumed by the child
    - less than 24 empty sachet counted
  - For all three groups (RUTF, MNP, control): having taken RUTF or MNP outside the study allocation (e.g. as part of measles treatment)
- Patient misses 3 or more scheduled visits
- Patient misses 2 or more consecutive scheduled visits
- Patient misses the last scheduled visit (6 months visit)
- Participation in other feeding programmes

4.3 Completed the study

Patients will be considered as having 'completed the study' if they have attended at least the first and last scheduled visit at 6 months.

4.4 Baseline drop-out

All patients enrolled who dropped out before the start of randomized treatment.

5 Statistical analysis

5.1 Analysis of baseline data

All available data at baseline will be analysed descriptively for each intervention group and in total. Results will be summarised as frequencies and percentages for nominal data; as means, standard deviations, and range for continuous data; and as medians, quartiles, and ranges for ordinal data.
Differences regarding baseline variables will be checked for clinical relevance between the three treatment groups.

The flow of participants will be described (e.g. number screened, recruited, randomized, emergency unblinding, etc.). Number and reasons for drop-out, lost-to-follow-up, or withdrawal will be presented. If possible respondents and non-respondent will be compared.

5.2 Compliance

Compliance to RUTF and MNP will be assessed through questionnaire and collection of returned supplement sachets.
Compliance is measured as the percentage of dispensed sachets returned empty and will be described by mean percentage (with range and standard deviation) for each treatment group.

5.3 Primary analysis of primary endpoint

The primary endpoint is “negative nutritional outcome” of a child within the 6 months follow-up period. The incidence rate (in formulae below referred to simply as ‘rate’) of “negative nutritional outcome” will be computed as the numbe
r of events divided by total observed person-time in each treatment group. As an “event” the first time a child reaches a negative nutritional outcome will be used. The primary analysis of the primary endpoint will be performed on the intention to treat population with no imputation of missing data.

In the primary analysis, RUTF will be compared to the control group
The hypotheses in this step are

H_0: $rate_{RUTF} = rate_{Control}$
vs.
H_1: $rate_{RUTF} \neq rate_{Control}$

where $rate_{RUTF}$= rate of “negative nutritional outcome” in the children in the RUTF group, and $rate_{Control}$= rate of “negative nutritional outcome” in the children in the control group.

The comparison of rates among the two treatment groups (RUTF vs. control) will be performed by a Poisson regression model, adjusted for nutritional status at enrolment (fixed effect) and will be performed within one model including the three intervention groups with contrasts for each two-group comparison. The result will be presented as rates for each intervention group, and a rate ratio for the pair-wise comparison (each with 95% confidence intervals).Tests will be two-sided with a significance level of 0.05.

5.4 Secondary analysis of the primary endpoint

5.4.1 Treatment group comparisons

Although the study is primarily powered for the comparison between the RUTF and control group the other groups will be compared as well, keeping in mind that the sample size might not be large enough to obtain significant results.
Since there are three interventional groups to be compared, a hierarchical test procedure will be used to account for multiplicity.

i) In a first (primary) step, RUTF will be compared to the control group which is described above as analysis of primary outcome
If the RUTF group is not significantly different at a 0.05 level (two-sided test), the hierarchical procedure is stopped (all following analyses will be considered explorative). If the RUTF group is significantly different at a 0.05 level, the hierarchical procedure is continued with step 2.

ii) In the second step, MNP will be compared to the control group
The hypotheses in this step are

H_0: $rate_{MNP} = rate_{Control}$
vs.
H_1: $rate_{MNP} \neq rate_{Control}$

where $rate_{MNP}$=rate of “negative nutritional outcome” in the children in the MNP group, and $rate_{Control}$=rate of “negative nutritional outcome” in the children in the control group.

If the treatment factor is not significant at a 0.05 level (two-sided test), the hierarchical procedure is stopped (all following analyses will be considered explorative). If the treatment factor is significant at a 0.05 level, the hierarchical procedure is continued with step iii).

iii) In the third step, RUTF will be compared to MNP (non-inferiority test).
The hypotheses in this step are

H_0: $rate_{RUTF}$ - $rate_{MNP}$ $> - \delta$
vs.
H_1: $rate_{RUTF}$ - $rate_{MNP}$ $\leq - \delta$

where $rate_{RUTF}$=rate of "negative nutritional outcome" in the children in the RUTF group, $rate_{MNP}$=rate of ""negative nutritional outcome" in the children in the MNP group, and δ=inferiority margin.

As non-inferiority margin, a δ of 2% (absolute value) will be used (i.e. allowing a slightly worse result in the MNP compared to the RUTF group). The third step will be tested at a 0.025 level (one-sided test), by a one-sided 97.5% confidence interval of the difference in rates between the RUTF and the MNP group.
The general problem of assay sensitivity in non-inferiority comparisons can be addressed in this study by the 3-arm design including a control group receiving no nutritional supplementation. Thus, superiority of the two nutritional interventions (RUTF and MNP) versus no intervention can be evaluated prior to the non-inferiority comparison.

These step-wise comparisons of rates among treatment groups will be performed by the same Poisson regression model, adjusted for nutritional status at enrolment (fixed effect) as described above, i.e. performed within one model including the three intervention groups with contrasts for each two-group comparison. The results will be presented as rates for each intervention group, and rate ratios for the pair-wise comparison (each with 95% confidence intervals).

5.4.2 Further adjustments

In case of relevant differences in baseline characteristics additional adjustment(s) for these factors will be performed within the model described in 5.4.1 to address potential confounding.

5.4.3 Subgroups

Stratified analysis will be done for three pair-wise comparisons (RUFT vs. control, MNP vs. control, RUTF vs. MNP (as described in 5.4.1) according to the following subgroups (measured at baseline):

- age (binary: <36 months, ≥36 months)
- gender
- breastfeeding practices (no, partial breast feeding)
- underlying illnesses (only malaria, only diarrhoea, only LRTI, 2 or more diseases)
- socio-economic status (low, moderate, high)
- food availability (binary or in tertile)
- length of illness before visiting MSF clinic (binary or in tertile)
- recruitment period (binary or in tertile)

To assess the presence of effect modification for these factors, models with interaction terms will be analysed.

5.4.4 Missing endpoint data

The amount, pattern, timing and distribution of missing data of the primary endpoint will be checked by a person blinded to treatment allocation. A decision based upon the result of this check will be made as to whether and how imputation of missing primary endpoint data will be performed as a sensitivity analysis on the ITT population for the analysis in 5.4.1.

5.4.5 Per protocol analysis

The analysis as described in 5.4.1 will be repeated for the per protocol population.

5.5 Analysis of secondary endpoints

All secondary endpoints (as defined in section 3.2) will be compared between the three treatment groups as described in 5.4.1 (p-values will be considered explorative) for the ITT and the PP populations. In addition to the Poisson regression model, logistic regression model, analysis of variance (ANOVA), analysis of covariance (ANCOVA), Cox proportional hazard model, and non-parametric analysis of variance will be used, according to the type of endpoint. In case of relevant differences in baseline characteristics additional adjustment(s) for these factors may be performed. Stratified analyses are planned for secondary endpoints regarding weight and weight change with respect to age, gender, and breast feeding.

5.6 Analysis of safety measures

The proportion of children with serious adverse events or serious adverse reactions will be presented by frequencies and percentages for each treatment group. The number of serious adverse events and serious adverse reactions will be presented by frequencies for each treatment group.

5.7 Joint analysis of data from other studies

Prior to this study a similar study (pilot) had been conducted in Katanga, DRC (length of study 28 days), and a similar study will be conducted in Goronyo, Nigeria (following the same protocol as the present study).

5.7.1 Pooled analysis I

It is planned to pool the data up to 28 days from the three studies (Katanga, Uganda, Nigeria) and analyse it jointly with adjustment for the study location by meta-analysis.

5.7.2 Pooled analysis II

In addition, the data from the present study (Uganda) and the study in Nigeria will be pooled and analysed jointly with adjustment for the study location by meta-analysis or by individually pooled data analysis.

6 Software

Software used will be SAS for Windows, Version 9.2 or higher (SAS Institute, Cary, NC, USA) and STATA, Version 11 (StataCorp, College Station, Texas, USA), and Review Manager (RevMan) Version 5.1. (Copenhagen: The Nordic Cochrane Centre, The Cochrane Collaboration, 2011).
